# Abrogating cholesterol esterification suppresses growth and metastasis of pancreatic cancer

**DOI:** 10.1038/onc.2016.168

**Published:** 2016-05-02

**Authors:** J Li, D Gu, S S-Y Lee, B Song, S Bandyopadhyay, S Chen, S F Konieczny, T L Ratliff, X Liu, J Xie, J-X Cheng

**Affiliations:** 1 Weldon School of Biomedical Engineering, Purdue University, West Lafayette, IN, USA; 2 Department of Pediatrics, Wells Center for Pediatric Research, IU Simon Cancer Center, Indiana University School of Medicine, Indianapolis, IN, USA; 3 Department of Biological Sciences, Purdue University, West Lafayette, IN, USA; 4 Department of Pathology and Laboratory Medicine, Indiana University School of Medicine, Indianapolis, IN, USA; 5 Center for Cancer Research, Purdue University, West Lafayette, IN, USA; 6 Department of Comparative Pathobiology, Purdue University, West Lafayette, IN, USA; 7 Department of Biochemistry, Purdue University, West Lafayette, IN, USA

## Abstract

Cancer cells are known to execute reprogramed metabolism of glucose, amino acids and lipids. Here, we report a significant role of cholesterol metabolism in cancer metastasis. By using label-free Raman spectromicroscopy, we found an aberrant accumulation of cholesteryl ester in human pancreatic cancer specimens and cell lines, mediated by acyl-CoA cholesterol acyltransferase-1 (ACAT-1) enzyme. Expression of ACAT-1 showed a correlation with poor patient survival. Abrogation of cholesterol esterification, either by an ACAT-1 inhibitor or by shRNA knockdown, significantly suppressed tumor growth and metastasis in an orthotopic mouse model of pancreatic cancer. Mechanically, ACAT-1 inhibition increased intracellular free cholesterol level, which was associated with elevated endoplasmic reticulum stress and caused apoptosis. Collectively, our results demonstrate a new strategy for treating metastatic pancreatic cancer by inhibiting cholesterol esterification.

## Introduction

Metastasis is the major cause of cancer-related mortality. Though localized tumors can often be treated by surgery or other therapies, treatment options for metastatic diseases are limited. Cancer metastasis has been revealed to be a multiple step process, including cancer cell migration, local invasion, intravasation, circulation through blood and lymph vessels, extravasation, survival and colonization in distant organs.^1, 2, 3^ Mediators identified in these processes have provided the basis for the development of therapies to target metastasis. Current therapeutic strategies for treating metastatic tumors mainly focus on targeting the adhesive molecules and extracellular proteases.^4^ However, these therapeutics have not been proven to be effective in clinical trials, partially owing to the various escape mechanisms used by the metastatic cancer cells.^2, 5, 6^ Thus, an unmet need exists to develop new therapeutic strategies for treating metastatic cancers.

Recent advances in cancer metabolism have unveiled many potential therapeutic targets for cancer treatment. Metabolic reprogramming, a strategy used by cancer cells to adapt to the rapid proliferation, is being recognized as a new hallmark of cancer.^7^ Substantial studies have found increased glycolysis, glutaminolysis, nucleotide and lipid synthesis in cancer cells.^7, 8, 9, 10^ Considering that altered metabolic pathways only happen in cancer cells but not in normal cells, targeting these pathways may provide cancer-specific treatments. A number of inhibitors of metabolic enzymes, such as glycolysis inhibitors, are under clinical trials as targeted cancer therapeutics.^11^

Of various metabolic pathways, lipid metabolism has been suggested to have an important role in cancer cell migration, invasion and metastasis.^12^ A recent study reported that surrounding adipocytes provide energy source for ovarian cancer cells to promote its rapid growth and metastasis.^13^ Blocking lipid *de novo* synthesis pathway has been shown to suppress tumor regrowth and metastasis after anti-angiogenesis treatment withdrawal.^14^ In parallel, lipolysis by the enzyme monoacylglycerol lipase was shown to regulate the fatty acid network, which promotes cancer cell migration, invasion and growth.^15^

Cholesterol, a critical component of the plasma membrane, is also implied to be correlated to cancer metastasis.^16^ It has been shown that prostate cancer bone metastases contain a high level of cholesterol.^17^ Modulation of cholesterol level in plasma membrane was shown to regulate the capability of cell migration.^18, 19^ Moreover, cholesterol-enriched lipid rafts were shown to have an essential role in cancer cell adhesion and migration.^20^ Mammalian cells obtain cholesterol either from *de novo* synthesis or from the uptake of low-density lipoprotein (LDL).^21^ Inside cells, excess free cholesterol is esterified and stored as cholesteryl ester (CE) in lipid droplets (LDs), which is mediated by acyl-CoA cholesterol acyltransferase (ACAT).^22^ Increased CE level has been reported in breast cancer,^23^ leukemia,^24^ glioma^25^ and prostate cancer.^26^ Despite these advances, the role of cholesterol esterification in cancer progression, especially in cancer metastasis, is not well understood.

In this article, we report a link between cholesterol esterification and metastasis in pancreatic cancer. Using stimulated Raman scattering (SRS) microscopy and Raman spectroscopy to map LDs stored inside single cells and analyze the composition of individual LDs, we identified an aberrant accumulation of CE in human pancreatic cancer specimens and cell lines. Abrogation of cholesterol esterification, either by inhibiting ACAT-1 enzyme activity or by shRNA knockdown of ACAT-1 expression, significantly reduced pancreatic tumor growth and metastasis in an orthotopic mouse model. Mechanistically, inhibition of cholesterol esterification disturbed cholesterol homeostasis by increasing intracellular free cholesterol level, which was associated with elevated endoplasmic reticulum (ER) stress and eventually led to apoptosis.

## Results

### Aberrant accumulation of CE in human pancreatic cancer tissues and cell lines, but not in normal counterparts

**Figure 1:**
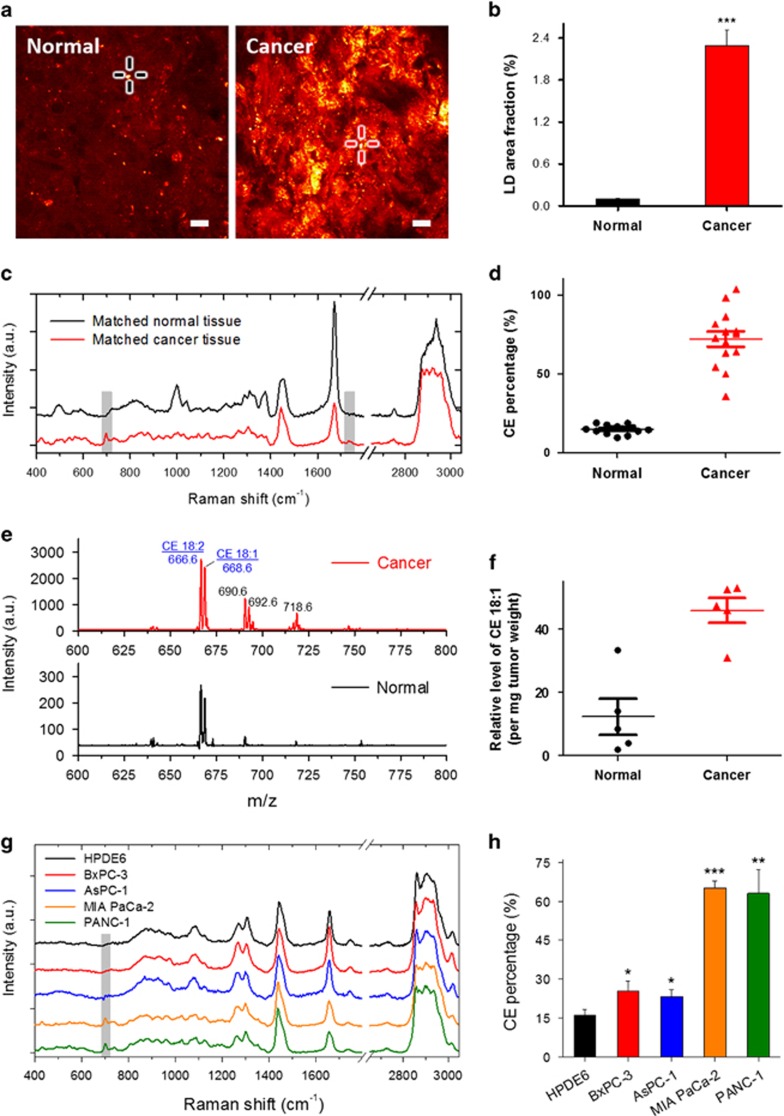
CE accumulation in human pancreatic cancer tissues and cell lines. (**a**) Representative SRS images of matched normal and cancerous human pancreatic tissue slices. Scale bar: 10 μm. (**b**) Quantitative analysis of area fraction of LDs out of the total cellular area based on SRS images. (**c**) Representative Raman spectra acquired from individual LDs crossed marked in images in A. The peak of cholesterol at 702 cm^−1^ and the peak of ester bond at 1742 cm^−1^ were highlighted in gray shade. The spectra were offset for clarity. (**d**) Quantitative analysis of CE percentage out of total lipids in 14 pairs of normal and cancer tissues based on Raman spectra. The bars represent means±s.e.m. (**e**) Representative mass spectra of CEs extracted from one pair of matched normal and cancer tissues. (**f**) Quantitative analysis of the amount of CE 18:1 in five pairs of normal and cancer tissues based on mass spectra. (**g**) Representative Raman spectra taken from individual LDs in human pancreatic cell lines. The cholesterol peaks at 702 cm^−1^ were highlighted in gray shade. (**h**) Quantitative analysis of CE percentage out of total lipids in cell lines. The data are shown as means+s.e.m.; *n*⩾10; *P<0.05, **P<0.01, ***P<0.001.

Using Raman spectromicroscopy, we mapped lipid distribution and analyzed the composition in individual LDs inside single cells. Cryo-sections of matched normal and cancerous human pancreatic tissues from same patients were used, of which the pathological status was confirmed by a pathologist. Totally, 14 pairs of matched normal and cancerous tissues were imaged and analyzed. Our SRS imaging revealed a much higher level of LD accumulation in cancerous pancreatic tissues than in normal tissues (Figure 1a). To make sure that the images were taken from the cancer cells, but not the stromal cells, histologically stained adjacent slides were used to identify the cancer cells. By quantitative analysis of the SRS images, we showed that the amount of LDs in cancer tissues is over 20 times higher than that in normal tissues (Figure 1b).

To assess the composition of LDs in cancer and normal tissues, we acquired Raman spectra from individual LDs inside the cells (Figure 1c). The LDs in pancreatic cancer cells contained high levels of CE, indicated by the characteristic ester bond vibration mode at 1742 cm^−1^ and the cholesterol ring vibration mode at 702 cm^−1^.^27^ Multiple Raman spectra from individual LDs in the same cells were recorded to ensure consistency (Supplementary Figure S1A). It is worth noting that spectra from normal tissues showed no peak at 702 cm^−1^ but a strong peak at 2930 cm^−1^, indicating high protein content in those LDs. Using emulsions mixed by cholesteryl oleate and glyceryl trioleate, we verified that height ratio of the Raman peak at 702 cm^−1^ to the peak at 1442 cm^−1^ (CH_2_ bending vibration mode) is linearly proportional to the molar percentage of CE (Supplementary Figure S1B). On the basis of this calibration curve, we found that the percentage of CE in pancreatic cancer tissues ranged from 60 to 95%, while the percentage in normal tissues was within 10~20% (Figure 1d).

To further validate our Raman spectral measurements, we used mass spectrometry to analyze all CE species extracted from the matched normal and cancer tissues. Two major forms of CEs in normal and cancer tissues were identified to be CE 18:1 (for example, cholesteryl oleate) and CE 18:2 (for example, cholesteryl linoleate) (Figure 1e). A quantitative analysis revealed a significant increase of both CE 18:1 (Figure 1f) and CE 18:2 (Supplementary Figure S1C) in cancer tissues compared with matched normal ones. Independently, increased levels of CE in cancer tissues were confirmed by a colorimetric assay (Supplementary Figure S1D). Next, we studied lipids in human pancreatic cell lines, including normal immortalized pancreatic epithelial cell line HPDE6, pancreatic cancer cell lines BxPC-3, AsPC-1, MIA PaCa-2 and PANC-1. Consistent with the human specimen data, SRS imaging (Supplementary Figure S1E) and Raman spectral analysis (Figure 1g) revealed higher CE levels in cancer cells than in normal cells (Figure 1h). Collectively, these data suggest that CE accumulation is a metabolic event that occurs in pancreatic cancer cells but not in normal cells.

### ACAT-1 mediates the accumulation of CE and positively correlates with poor survival in pancreatic cancer patients

**Figure 2:**
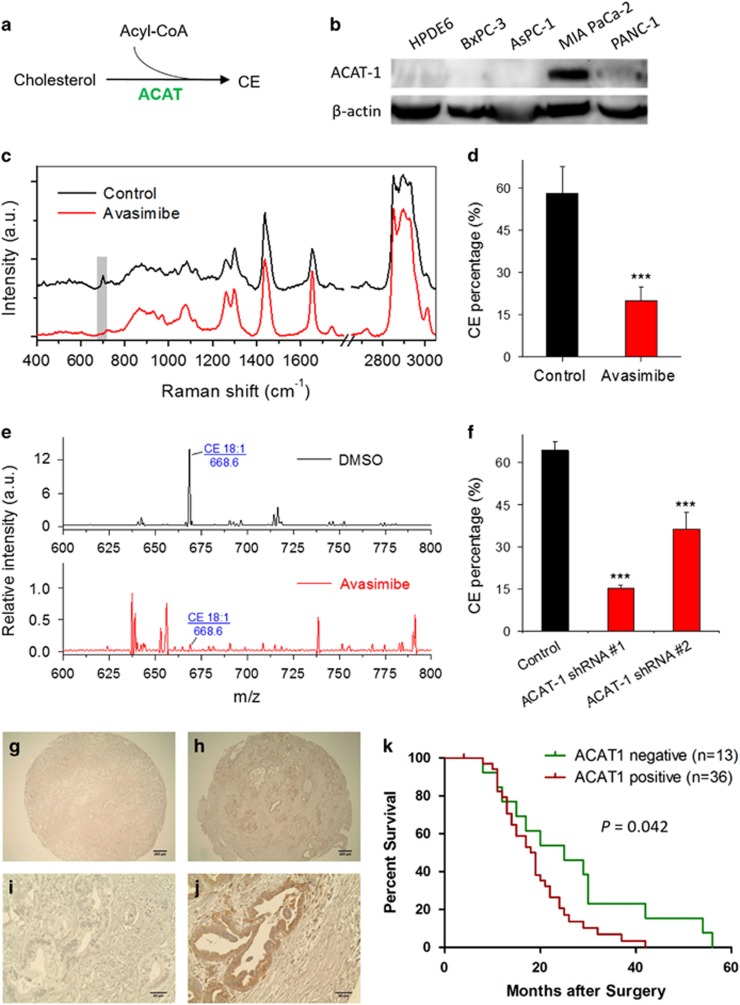
CE accumulation in pancreatic cancer is mediated by ACAT-1 and expression of ACAT-1 correlates with poor patient survival. (**a**) ACAT catalyzes the synthesis of CE from free cholesterol and fatty acyl-CoA. (**b**) Expression levels of ACAT-1 in pancreatic cell lines measured by immunoblotting. (**c**) Representative Raman spectra of MIA PaCa-2 cells treated with DMSO (control) or 10 μM avasimibe for 2 days. The peak at 702 cm^−1^ was highlighted in gray shade. (**d**) Quantification of CE level after avasimibe treatment. (**e**) Representative mass spectra of CEs extracted from MIA PaCa-2 cells treated with DMSO (control) or 10 μM avasimibe for 2 days. (**f**) Quantification of CE level in MIA PaCa-2 cells transfected with control shRNA or two ACAT-1-specific shRNAs. The quantification data are shown as means+s.e.m.; *n*⩾10; ***P<0.001. (**g**–**j**) Immunohistochemistry of ACAT-1 on human pancreatic cancer tissue array. (**g**) a represents ACAT-1 negative samples, and (**h**) represents ACAT-1-positive samples. (**i** and **j**) Zoom in images from (**g**) and (**h**), respectively. (**k**) ACAT-1 expression is correlated with poor patient survival rate. *N*=13 for ACAT-1-negative group, and *N*=36 for ACAT-1-positive group. *P*=0.042 was determined by Log Rank test.

In mammalian cells, synthesis of CE from free cholesterol is mainly catalyzed by acyl coenzyme A: cholesterol acyltransferase (ACAT) enzymes, ACAT-1 and ACAT-2 (Figure 2a), of which the latter one is primarily expressed in intestinal mucosal cells in human.^21, 22^ To test which isoform contributes to the CE accumulation in pancreatic cancers, we first examined the expression level of ACAT-1 and ACAT-2 in matched normal and cancer human pancreatic tissues. ACAT-1 expression was shown positive in cancer tissues, but marginal in the normal counterparts (Supplementary Figure S2A). In contrast, expression of ACAT-2 had no differences between normal tissues and the matched cancer tissues (Supplementary Figure S2A), implying that ACAT-2 may not be critical for CE generation in pancreatic cancer cells. Higher expression levels of ACAT-1 were also observed in MIA PaCa-2 and PANC-1 cells than HPDE6, BxPC-3 and AsPC-1 cells (Figure 2b), which are positively correlated with their CE levels. Then, a potent inhibitor of ACAT, avasimibe, was applied to pancreatic cancer cells. As expected, inhibition of ACAT by avasimibe effectively blocked CE accumulation in Mia PaCa-2 cells (Figures 2c and d) and PANC-1 cells (Supplementary Figure S2B). Reduction of CE by avasimibe was confirmed by mass spectrometry, which also revealed that the principal form of CE in Mia PaCa-2 cells (Figure 2e) and PANC-1 cells (Supplementary Figure S2C) was CE 18:1 (for example, cholesteryl oleate). As avasimibe is known to inhibit both ACAT-1 and ACAT-2,^28^ we conducted knockdown of ACAT-1 by specific shRNA (Supplementary Figure S2D). As expected, ACAT-1 shRNA effectively suppressed CE accumulation in pancreatic cancer cells (Figure 2f, Supplementary Figure S2E). These evidences collectively support ACAT-1 is the major isoform that promotes the CE accumulation in pancreatic cancers.

The overexpression of ACAT-1 in cancer tissues suggests a potential role of ACAT-1 in pancreatic cancer progression. To correlate ACAT-1 expression with patient outcome, we performed immunohistochemistry of ACAT-1 in a pancreatic cancer tissue array. Out of the 49 pieces of cancer tissues, 13 were identified as ACAT-1-negative (Figures 2g and i) and 36 were positive (Figures 2h and j). Importantly, the ACAT-1-negative patients survived significantly longer than the ACAT-1-positive patients (*P*=0.042, Log Rank test), with a median survival time of 25 months for ACAT-1-negative patients and 17 months for ACAT-1-positive patients (Figure 2k). These clinical data suggest that ACAT-1 expression is a potential prognosis marker for pancreatic cancer.

### CE accumulation in pancreatic cancer is regulated by PTEN and mediated by both *de novo* cholesterol synthesis and LDL uptake

**Figure 3:**
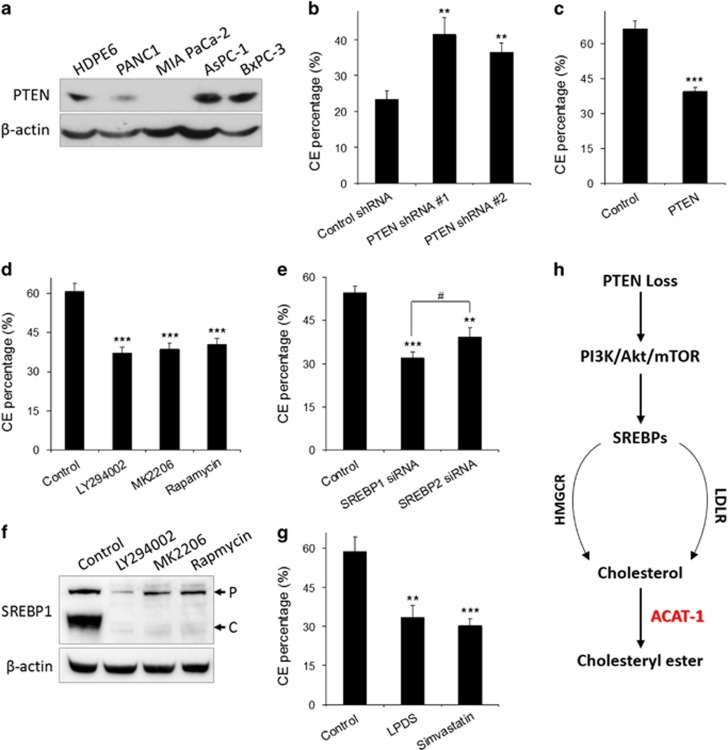
CE level in pancreatic cancer is regulated by PTEN and mediated by *de novo* cholesterol synthesis and LDL uptake. (**a**) Expression levels of PTEN in human pancreatic cell lines measured by immunoblotting. (**b**) CE measurement in AsPC-1 cells transfected with scramble (control) shRNA or two PTEN-specific shRNAs. (**c**) CE measurement in MIA PaCa-2 cells transfected with empty vector (control) or pLKO plasmid containing wild-type PTEN. (**d**) CE measurement in MIA PaCa-2 cells treated with DMSO (control), PI3K inhibitor (50 μM LY294002), Akt inhibitor (10 μM MK2206) or mTOR inhibitor (100 nM Rapamycin) for 3 days. (**e**) CE measurement in MIA PaCa-2 cells transfected with scramble siRNA (control), SREBP1-specific siRNA or SREBP2 specific siRNA. (**f**) Expression levels of SREBP1 in MIA PaCa-2 cells treated with 50 μM LY294002, 10 μM MK2206 or 100 nM Rapamycin for 2 days. P: precursor form; C: cleaved form. (**g**) CE measurement in MIA PaCa-2 cells cultured in lipodeficient serum (LPDS) supplemented medium or treated with 10 μM simvastatin for 2 days. The CE quantification data are shown as means+s.e.m.; *n*⩾10; ^#^*P*<0.05, *P<0.05, **P<0.01, ***P<0.001. (**h**) A diagram showing the molecular signaling regulating CE accumulation in pancreatic cancer.

Although higher levels of CE were found in pancreatic cancer cell lines, our results showed that CE levels varied in different cell lines. Specifically, MIA PaCa-2 and PANC-1 cells had much higher levels of CE than AsPC-1 and BxPC-3 cells. As PTEN loss has been shown to drive the CE accumulation in prostate cancer,^26^ we asked whether the CE level in pancreatic cancer cell lines is related to PTEN expression. We performed immunoblotting analysis of PTEN and found much higher levels of PTEN in CE-low HPDE6, AsPC-1 and BxPC-3 cells, but lower levels of PTEN in CE-rich MIA PaCa-2 and PANC-1 cells, suggesting a negative correlation between PTEN and CE level in pancreatic cancer cells (Figure 3a). To determine whether PTEN regulates CE accumulation, we conducted knockdown of PTEN by specific shRNA in AsPC-1 cells (Supplementary Figure S3A) and induced overexpression of wild-type PTEN in MIA PaCa-2 cells (Supplementary Figure S3B). As expected, knockdown of PTEN by shRNA significantly increased CE levels in AsPC-1 cells (Figure 3b), while overexpression of wild-type PTEN significantly reduced CE in MIA PaCa-2 cells (Figure 3c). The role of PTEN in regulating CE levels is speculated to be through the PI3K/Akt/mTOR pathway, which is downstream of PTEN.^29^ Indeed, inhibition of PI3K, Akt and mTOR by inhibitors significantly reduced CE levels in MIA PaCa-2 cells (Figure 3d). It is also known that mTOR complex regulates lipogenesis through controlling the activity of Sterol Regulatory Element-Binding Proteins (SREBPs).^30^ Consistently, knockdown of SREBP1 and SREBP2 by siRNAs significantly reduced CE levels in MIA PaCa-2 cells (Figure 3e). The effect of SREBP1 knockdown is more prominent than SREBP2 knockdown, suggesting that SREBP1 is more directly involved in CE accumulation in pancreatic cancer. Furthermore, knockdown of PTEN increased the expression of mature form SREBP1 (Supplementary Figure S3C). Meanwhile, overexpression of PTEN (Supplementary Figure S3D) or inhibition of PI3K/Akt/mTOR by inhibitors (Figure 3f) significantly reduced the expression of mature form SREBP1. These data collectively demonstrate that CE accumulation is regulated by PTEN activity and subsequent activation of the PI3K/Akt/mTOR/SREBP signaling pathway.

Cancer cells obtain cholesterol either from *de novo* synthesis or by uptake of extracellular LDL.^21^ To investigate which pathway contributes to CE accumulation in pancreatic cancer, we used simvastatin, a specific inhibitor of 3-hydroxy-3-methylglutaryl coenzyme A (HMG-CoA) reductase, the rate-limiting enzyme in the cholesterol synthesis pathway, to block the *de novo* synthesis pathway. We also applied lipodeficient serum to deplete the extracellular cholesterol carried in LDLs. Either lipodeficient serum supplementation or HMG-CoA reductase inhibition significantly reduced the CE level (Figure 3g), indicating that both *de novo* synthesis and LDL uptake pathways contribute to CE accumulation in pancreatic cancer cells. Avasimibe treatment also significantly reduced the uptake of LDL as shown by fluorescence imaging (Supplementary Figure S3E) and quantitative analysis of intracellular DiI-labeled LDL (Supplementary Figure S3F), indicating that LDL uptake is a tightly controlled process in response to the regulation of cholesterol hemostasis. Together, our results indicate that CE accumulation in pancreatic cancer arises from both *de novo* synthesis and LDL uptake, and is mediated by the ACAT-1 enzyme (Figure 3h).

### Blocking cholesterol esterification suppresses pancreatic cancer growth and metastasis

**Figure 4:**
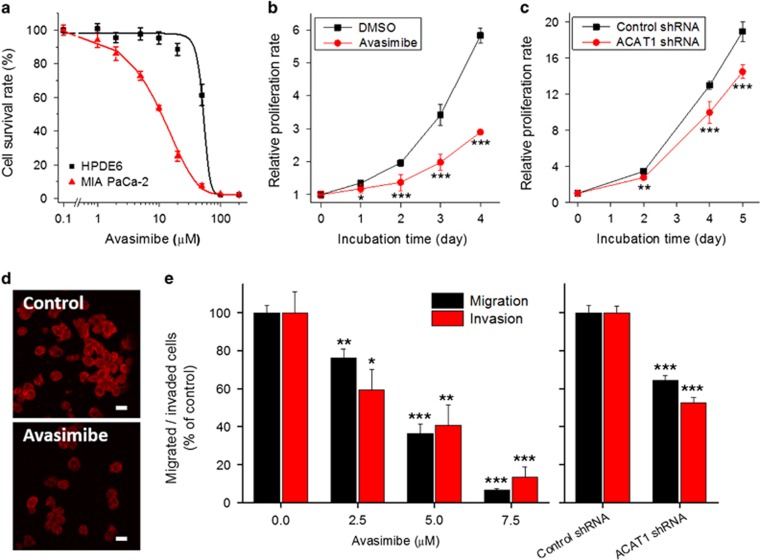
Inhibition of cholesterol esterification reduced pancreatic cancer cell proliferation, migration and invasion *in vitro*. (**a**) Cell viability assay of HPDE6 and MIA PaCa-2 cells treated with avasimibe for 3 days. The data were fitted with a dose–response function using software Origin8.5. (**b**) Cell proliferation assay of MIA PaCa-2 cells treated with DMSO or 10 μM avasimibe. (**c**) Cell proliferation assay of MIA PaCa-2 cells stably transfected with control shRNA or ACAT-1 shRNA. For cell viability or proliferation assay, data are shown as means±s.d.; *n*=6; *P<0.05, **P<0.01, ***P<0.001. (**d**) Representative images of MIA PaCa-2 cells migrated through Transwell membrane. The cells were treated with DMSO or 2.5 μM avasimibe, stained with 10 μg/ml PI for 30 min. Scale bar: 20 μm. (**e**) Quantification of the number of migrated and invaded cells treated with avasimibe at 0, 2.5, 5 and 7.5 μM or stably transfected with control shRNA or ACAT-1 shRNA. Cell number was counted using ImageJ cell counter function. The data are shown as means+s.e.m.; n⩾6; *P<0.05, **P<0.01, ***P<0.001.

Considering that CE accumulation is a cancer-specific event, we further tested whether cholesterol esterification could be a potential target for cancer therapy. By using avasimibe, a potent inhibitor of ACAT-1, we found that pancreatic cancer cells MIA PaCa-2 and PANC-1 were much more sensitive to ACAT-1 inhibition than normal HPDE6 cells (Figure 4a, Supplementary Figure S4A). The IC50 of avasimibe for MIA PaCa-2, PANC-1 and HPDE6 are 11.03, 23.58 and 52.81 μM, respectively. Inhibition of ACAT-1 by avasimibe at 10 μM significantly reduced the proliferation rate of MIA PaCa-2 (Figure 4b) and PANC-1 cells (Supplementary Figure S4B). To confirm that the anti-cancer effect of avasimibe is specific to ACAT-1 inhibition, knockdown of ACAT-1 by specific shRNA was performed. As predicted, MIA PaCa-2 cells with ACAT-1 knockdown showed a much-reduced proliferation rate (Figure 4c). Using the Transwell method, we further performed cell migration and invasion assays. Inhibition of ACAT-1, either by avasimibe or shRNA knockdown, significantly reduced MIA PaCa-2 cell migration and invasion rates (Figures 4d and e). Together, these results show that cancer cells are highly sensitive to blockage of cholesterol esterification.

**Figure 5:**
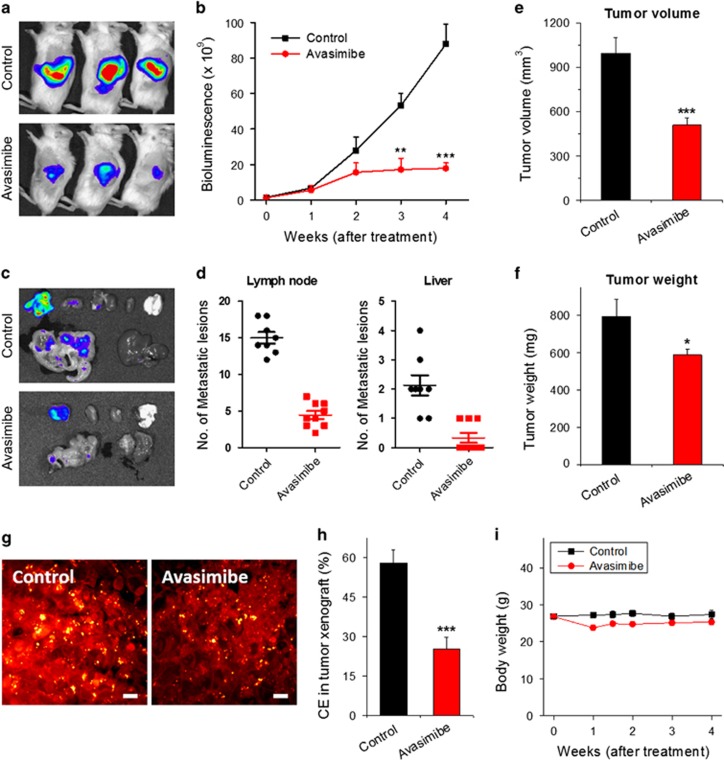
Therapeutic effect of avasimibe in an orthotopic mouse model of pancreatic cancer. (**a**) Representative IVIS images of mice treated with vehicle (control) or avasimibe at a dosage of 15 mg/kg per day for 4 weeks. (**b**) Tumor growth curve quantified by total intensity of IVIS imaging. (**c**) Representative IVIS images of metastatic lesions in organs (from left to right, top to bottom: pancreas, spleen, kidney, lung/heart, intestine and liver). (**d**) Number of metastatic lesions in lymph node and liver. The bars represent the means. (**e**, **f**) *Ex vivo* measurement of tumor volume and weight. (**g**) Representative SRS images of tumor tissue slices. Scale bar: 10 μm. (**h**) CE measurement in tumor tissues. (**i**) Monitoring of body weight over time. The data are shown as means +/± s.e.m.; *n*=8 for control group, *n*=9 for avasimibe group; *P<0.05, **P<0.01, ***P<0.001.

Next, we deployed a well-established orthotopic mouse model of pancreatic cancer^31^ to validate the anti-cancer effect of ACAT-1 inhibitor *in vivo*. MIA PaCa-2 cells with luciferase and mCherry expression were orthotopically injected into the pancreas. Tumor growth was monitored weekly by *In Vivo* Imaging System (IVIS) imaging by detecting the luminescence signal *in vivo*. Avasimibe was prepared using a water-soluble formulation by complexing with human serum albumin^32^ and intraperitoneally injected at a dose of 15 mg/kg per day. The results showed that avasimibe treatment for 4 weeks remarkably suppressed tumor size, as indicated by luminescence signal intensity (Figure 5a) and largely reduced tumor growth rate (Figure 5b). Metastatic lesions in lymph nodes and distant organs (for example, liver, spleen and lung) were also assessed by IVIS imaging at the end point of the study (Figure 5c). Much higher number of metastatic lesions in lymph nodes were detected in the control group (15.0±2.2, *n*=8) than the avasimibe-treated group (4.4±1.7, *n*=9). Each mouse in the control group showed, at least, one metastatic lesion in the liver. In contrast, only three mice in the avasimibe-treated group showed single lesion in liver (Figure 5d). *Ex vivo* measurement of tumor volume (Figure 5e) and tumor weight (Figure 5f) further confirmed that avasimibe reduced tumor size and weight. SRS imaging showed a decrease of the number of LDs (Figure 5g), and Raman spectral analysis verified a significant reduction of CE level in LDs (Figure 5h), suggesting that avasimibe acted by blocking cholesterol esterification. Moreover, avasimibe did not induce body weight loss (Figure 5i). The pathological assessment also confirmed no apparent organ toxicity in liver, kidney, lung and spleen, as shown by hematoxylin and eosin staining (Supplementary Figure S5).

**Figure 6:**
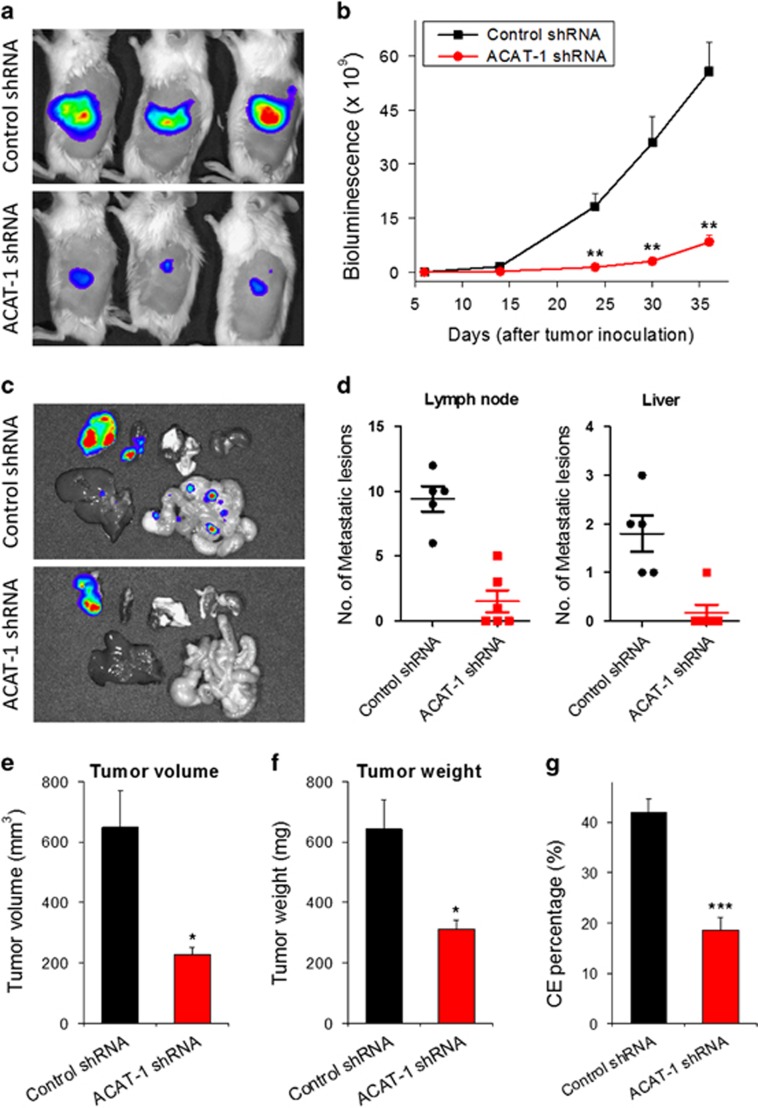
ACAT-1 knockdown suppressed tumor growth and metastasis in an orthotopic mouse model of pancreatic cancer. (**a**) Representative IVIS images of mice 5 weeks after implantation of MIA PaCa-2^luc/mCherry^ cells stably transfected with control shRNA or ACAT-1 shRNA. (**b**) Tumor growth curve quantified by total intensity of IVIS imaging. (**c**) IVIS images of metastatic lesions in organs (from left to right: pancreas, spleen, lung/heart, kidney, liver and intestine). (**d**) Number of metastatic lesions in lymph node and liver. The bars represent the means. (**e**, **f**) *Ex vivo* measurement of tumor volume and tumor weight. (**g**) CE measurement in the tumor tissues. ***P<0.001. The data are shown as means+s.e.m.; *n*=5 for control shRNA group, *n*=6 for ACAT-1 shRNA group; *P<0.05, **P<0.01, ***P<0.001.

To testify that the anti-cancer effect of avasimibe is based on ACAT-1 inhibition, an MIA PaCa-2^luc/mCherry^ cell line with stable knockdown of ACAT-1 was generated and applied to the orthotopic mouse model. After 5 weeks of tumor cell inoculation, the ACAT-1 knockdown cells developed significantly smaller tumors compared with the ACAT-1 wild-type cells (Figure 6a). Tumor growth was dramatically suppressed with ACAT-1 knockdown (Figure 6b) while no obvious loss in body weight was observed (Supplementary Figure S6A). As expected, metastatic lesions in lymph nodes and distant organs (for example, liver, spleen and lung) were suppressed by ACAT-1 knockdown (Figure 6c). Metastases in lymph nodes were developed in each mouse of ACAT-1 wild-type group (9.4±2.2, *n*=5), but only occurred in three mice of the knockdown group (1.5±2.1, *n*=6). Similarly, metastatic lesions in liver were found in each mouse of ACAT-1 wild-type group, but only occurred in one mouse in the knockdown group (Figure 6d). *Ex vivo* measurement of tumor volume and weight confirmed the tumor suppressing effect of ACAT-1 knockdown (Figures 6e and f). The slight decrease in total lipid amount (Supplementary Figure S6B) and significant reduction of CE level (Figure 6g) in the tumor tissues were confirmed by SRS imaging and Raman spectral analysis, respectively. Collectively, these data demonstrate the therapeutic potential of ACAT-1 inhibition for pancreatic cancer treatment.

### Inhibition of ACAT-1 induces ER stress and apoptosis in pancreatic cancer

**Figure 7:**
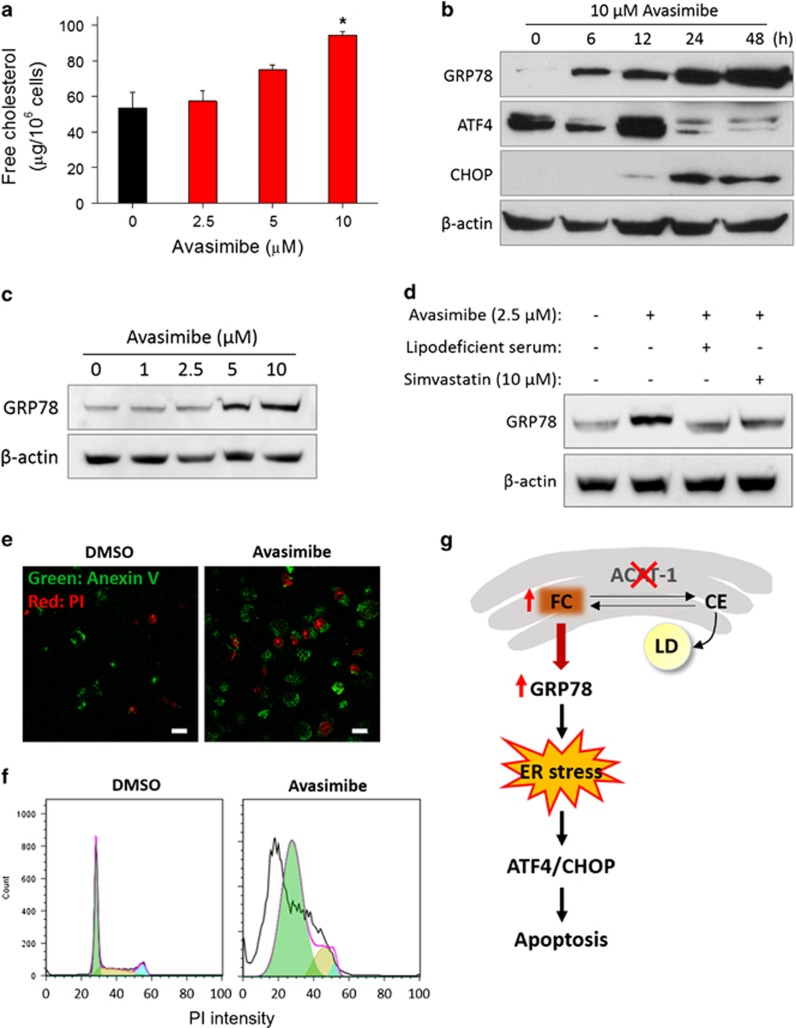
ACAT-1 inhibition increased cellular free cholesterol, induced ER stress and apoptosis. (**a**) Free cholesterol level measurement in MIA PaCa-2 cells treated with avasimibe at 0, 2.5, 5 and 10 μM for 2 days. The data are shown as means+s.e.m.; *n*=3; *P<0.05. (**b**) Immunoblotting of ER stress markers in MIA PaCa-2 cells at 0–48 h after treated with 10 μM avasimibe. (**c**) Expression level of GRP78 increased with avasimibe treatment dose-dependently. (**d**) Expression level of GRP78 at indicated treatment conditions. (**e**) Apoptosis assay by annexin V (green) and propidium iodide (PI, red) staining in MIA PaCa-2 cells treated with DMSO or 10 μM avasimibe for 2 days. Scale bar: 10 μm. (**f**) Cell cycle analysis by flow cytometry in MIA PaCa-2 cells treated with DMSO or 10 μM avasimibe for 2 days. (**g**) Diagram showing the mechanism of ACAT-1 inhibition induced cell toxicity.

The ACAT-1 enzyme esterifies free cholesterol to its esterified form, which can be stored in the LDs for maintenance of cholesterol homeostasis. We hypothesized that cholesterol esterification provides a way to minimize the cytotoxicity of excess free cholesterol caused by increased *de novo* cholesterol synthesis and LDL uptake. As we anticipated, free cholesterol levels gradually increased with avasimibe treatment from low to high concentrations (Figure 7a). Increased free cholesterol was also detected in the mouse pancreatic tumor tissues treated with avasimibe (Supplementary Figure S7A). Increased intracellular free cholesterol levels have been reported to be cytotoxic in macrophages by inducing ER stress and subsequent apoptosis.^33^ To test whether avasimibe treatment was associated with ER stress in pancreatic cancer cells, several ER stress markers were used, including 78 kDa glucose-regulated protein (GRP78), activating transcription factor 4 (ATF4) and C/EBP homologous protein (CHOP).^34^ Immunoblotting showed that GRP78 expression level gradually increased over time after avasimibe treatment, indicating the release of ER chaperone GRP78.^34^ The release of GRP78 activated subsequent unfolded protein response pathway, leading to an increase of transcription factor ATF4 within 12 h after treatment. ATF4 further induced expression of pro-apoptotic factor CHOP, expression of which appeared after 12-h treatment and increased from 12 to 48 h (Figure 7b). We further quantitated the extent of ER stress in MIA PaCa-2 cells treated with avasimibe from low to high concentrations, as indicated by a gradual increase of GRP78 (Figure 7c). Increased GRP78 expression was also observed in MIA PaCa-2 cells upon ACAT-1 knockdown (Supplementary Figure S7B). To demonstrate the association between ACAT-1 inhibition induced ER stress and elevated free cholesterol level, lipodeficient serum, which removes exogenous cholesterol, or simvastatin, which blocks cholesterol *de novo* synthesis, was applied to cells treated with avasimibe. It was shown that both lipodeficient serum and simvastatin reduced the GRP78 level (Figure 7d), partially rescued the cells from ER stress induced by avasimibe.

We further demonstrate that ACAT-1 inhibition-induced ER stress led to apoptosis of cancer cells. Using annexin V/propidium iodide staining (Figure 7e) and cell cycle analysis by flow cytometry (Figure 7f), we found that the number of apoptotic cells largely increased in avasimibe-treated MIA PaCa-2 cells. Increased apoptotic cells were also observed in avasimibe-treated tumor tissues compared with control tumor tissues, indicated by TUNEL assays (Supplementary Figures S7C and D). Taken together, ACAT-1 inhibition induced an increase of intracellular free cholesterol, ER stress and apoptosis in pancreatic cancer cells (Figure 7g).

## Discussion

In this study, we revealed a link between CE accumulation and pancreatic cancer metastasis. Accumulation of CE via ACAT-1 provides a mechanism to keep high metabolic activity and avoid toxicity from excess free cholesterol. Previously, CE has been reported in breast cancer,^23^ leukemia,^24^ glioma^25^ and prostate cancer.^26^ Inhibition of cholesterol esterification was shown to suppress tumor growth or cancer cell proliferation.^24, 25, 26^ Here, we demonstrate that inhibition of cholesterol esterification can be used to treat metastatic pancreatic cancer.

Cholesterol is an essential lipid having important roles in membrane construction, hormone production and signaling.^21^ Aberrant cholesterol metabolism is known to be associated with cardiovascular diseases and cancers.^35, 36^ Statins, inhibitors of HMG-CoA reductase, have been explored as potential therapies for pancreatic cancer.^37^ However, statins were not associated with a reduced risk of pancreatic cancer in clinical trials.^38^ One possible reason is that HMG-CoA reductase is also required for downstream protein prenylation, a critical process for protein activation.^39^ Thus, the effect of statin is not just inhibiting cholesterol synthesis, but also other pathways which may render toxicity to normal cells. This non-specific toxicity is a possible reason for the limited anti-cancer outcome of statin in clinical trials.

Our study identified cholesterol esterification as a novel target for suppression of pancreatic cancer proliferation and metastasis. Inhibitors of ACAT-1 are expected to have great value as cancer-targeting therapeutics, as CE accumulation only occurs in cancer tissues or cell lines. Our animal studies with avasimibe treatment showed no adverse effect to the animals at a dosage of 15 mg/kg. More importantly, modulation of cholesterol esterification suppressed not only tumor growth but also tumor metastasis. These results are expected to stimulate further biological studies to fully appreciate the role of cholesterol metabolism in cancer initiation and progression. As CE accumulation happens in several types of aggressive cancer, blocking cholesterol esterification could be pursued as a therapeutic strategy for other types of cancers. By combining with existing chemotherapies, such as gemcitabine, we believe this metabolic treatment possesses high possibilities to extend patients' survival time by retarding cancer progression and metastasis.

The molecular mechanism that links CE accumulation to cancer aggressiveness needs further studies. One possible mechanism is that cholesterol esterification keeps signaling pathways active by maintaining a low free cholesterol environment. One of the possible targets is the caveolin-1 signaling pathway. Caveolin-1, a regulator of cellular cholesterol homeostasis, is considered as a marker for pancreatic cancer progression.^11^ Particularly, a promoting role of caveolin-1 in pancreatic cancer metastasis has been reported.^40^ Our preliminary studies showed ACAT-1 inhibition reduced the expression level of SREBP1, caveolin-1 and phosphorylated ERK1/2 (unpublished data). The effect on caveolin-1 is probably mediated by SREBP1, which senses the intracellular cholesterol homeostasis.^41^ Meanwhile, caveolin-1 may have an important role in mediating the action of SREBP1 on MAPK pathways,^42, 43^ which are known to have essential roles in cancer cell metastasis.^44^ Therefore, it is possible that increased free cholesterol level induced by ACAT-1 inhibition inactivates SREBP1, leading to downregulation of caveolin-1/MAPK pathway, which contributes to the reduced cancer aggressiveness.

Besides the caveolin-1/MAPK signaling, other possibilities include the potential alteration of the membrane composition, such as lipid rafts, by ACAT-1 inhibition. Lipid rafts are known to provide platforms for multiple cellular signaling pathways.^20^ Thus, modulation of cholesterol metabolism is likely to have more profound effects via other signaling pathways. Future studies are needed to fully elucidate the molecular mechanism.

## Materials and methods

### Human pancreatic tissue specimens

This study was approved by Institutional Review Board. Frozen specimens of human pancreatic tissues were obtained from Indiana University Simon Cancer Center Solid Tissue Bank. Totally, 14 pairs of matched normal and cancerous tissues were collected. For each tissue specimen, pairs of adjacent tissue slices were prepared to be used. One slide remained unstained for spectroscopic imaging and the other stained with hematoxylin and eosin for pathological examination by a pathologist. Paraffin-embedded human pancreatic cancer tissue array was generated in IU School of Medicine. The use of human tissue was approved by the Institutional Review Boards of Indiana University.

### Cell lines and chemicals

Immortalized human pancreatic duct epithelial cell line HPDE6 and human pancreatic cancer cell line AsPC-1, BxPC-3, MIA PaCa-2 and PANC-1 were obtained from the American Type Culture Collection (ATCC). All cells were cultured at 37 °C in a humidified incubator with 5% CO_2_ supply. Cells were grown in the following media: Keratinocyte Serum Free Medium (Invitrogen, Carlsbad, CA, USA) supplemented with 30 μg/ml BPE and 0.2 ng/ml rEGF for HPDE6 cell; DMEM high glucose (Invitrogen) supplemented with 10% FBS for PANC-1 cell; RPMI 1640 (Invitrogen) supplemented with 10% FBS for AsPC-1, BxPC-3 and MIA PaCa-2 cells. MIA PaCa-2 cells with stable expression of luciferase and mCherry fluorescent protein was obtained from *In Vivo* Therapeutics Core at Indiana University Simon Cancer Center (Indiana University, IN) and grown in DMEM supplemented with 10% FBS.

Chemicals including cholesteryl oleate, glyceryl trioleate and simvastatin were purchased from Sigma–Aldrich (St Louis, MO, USA). Avasimibe used *in vitro* and *in vivo* studies were purchased from Selleckchem.com. Human LDL was purchased from Creative Laboratory Products (Indianapolis, IN, USA) and conjugated with DiI by the authors. Lipoprotein-deficient Serum was purchased from Biomedical Technologies (Ward Hill, MA, USA).

### *In vivo* study in orthotopic mouse model

All animal experiments were conducted following protocols approved by Purdue Animal Care and Use Committee (PACUC). Four- to 6-week-old male NOD/scid/IL2Rγ^null^ (NSG) mice were purchased from *In Vivo* Therapeutics Core at Indiana University Simon Cancer Center (Indiana University, IN) under a Material Transfer Agreement with Jackson Laboratories, Inc. Orthotopic mouse model of pancreatic cancer was established following a previously described protocol.^31^ MIA PaCa-2 cells with stable expression of luciferase and mCherry were collected and suspended at a concentration of 10 × 10^6^ cells/ml. A total of 5 × 10^5^ tumor cells in 50 μl media was directly injected into the pancreas of NSG mice. After recovery from surgery, tumor growth was monitored by bioluminescent imaging using IVIS in Bindley Bioscience Center at Purdue.

Mice were randomly divided into two groups. The group size was estimated based on prior studies.^26^ For the treatment with avasimibe at 15 mg/kg, intraperitoneal injection was used on a daily base, starting 1 week after tumor cell implantation. After treatment for 4 weeks, all the mice were killed. Tumors and metastatic lesions in the abdominal cavity, lymph nodes, liver, spleen, kidney, lung and heart were visualized with IVIS. Tumor volume and weight were measured *ex vivo*. Histological examination was performed by a pathologist to tumor and organ tissue slides after hematoxylin and eosin staining. The data analysis was validated by a second blinded author independently.

### Label-free Raman spectromicroscopy

Label-free Raman spectromicroscopy, including SRS microscopy, coherent anti-stokes Raman scattering microscopy and spontaneous Raman spectroscopy, was performed on unstained tissue slices (~15 μm) or cells without any labeling. Details of experimental procedures and data analysis are described in the Supplementary Materials and Methods.

### Statistical analysis

One-way analysis of variance or Student's *t*-test were used for comparisons between groups based on an assumption of normal distribution. Results were represented as means +/± s.e.m. or as specified. Kaplan–Meier survival curves were generated using SPSS. Significant differences were considered at *P<0.05, **P<0.01 and ***P<0.001.
