# Supplementary material for: Abrogating cholesterol esterification suppresses growth and metastasis of pancreatic cancer
Source: Oncogene. 2016 May 2;35(50):6378–88. doi: 10.1038/onc.2016.168 (PMC5093084; doi:10.1038/onc.2016.168)
Supplement: Supplementary Information [file onc2016168x1.docx]

**Supplementary information**

**Abrogating Cholesterol Esterification Suppresses Growth and Metastasis of Pancreatic Cancer**

Junjie Li^1^, Dongsheng Gu^2^, Steve Seung-Young Lee^1^, Bing Song^1^, Shovik Bandyopadhyay^3^, Shaoxiong Chen^4^, Stephen F. Konieczny^3, 7^, Timothy L. Ratliff ^5, 7^, Xiaoqi Liu^6, 7^, Jingwu Xie^2*^, Ji-Xin Cheng^1, 7*^

^1^ Weldon School of Biomedical Engineering, Purdue University, West Lafayette, IN 47907

^2^ Department of Pediatrics, Wells Center for Pediatric Research, IU Simon Cancer Center, Indiana University School of Medicine, Indianapolis, IN 46202

^3^ Department of Biological Sciences, Purdue University, West Lafayette, IN 47907

^4^ Department of Pathology and Laboratory Medicine, Indiana University School of Medicine, Indianapolis, IN 46202

^5^ Department of Comparative Pathobiology, Purdue University, West Lafayette, IN 47907

^6^ Department of Biochemistry, Purdue University, West Lafayette, IN 47907

^7^ Center for Cancer Research, Purdue University, West Lafayette, IN 47907

^*^ Corresponding authors: Ji-Xin Cheng, Weldon School of Biomedical Engineering, Purdue University, 206 S. Martin Jischke Dr., West Lafayette, IN 47907. Phone: 765-494-4335; Fax: 765-496-1912; Email: [jcheng@purdue.edu](mailto:jcheng@purdue.edu) or Jingwu Xie, Department of Pediatrics, Wells Center for Pediatric Research, IU Simon Cancer Center, Indiana University School of Medicine, 1044 W. Walnut St., Indianapolis, IN 46202. Phone: 317-278-3999; Fax: 317-274-8046; Email: [jinxie@iupui.edu](mailto:jinxie@iupui.edu).

***Running title:*** ACAT-1 is a therapeutic target for pancreatic cancer

**Supplementary Materials and Methods**

**Label-free Raman spectromicroscopy**

SRS imaging was conducted using a femtosecond laser source (Chameleon Vision, Coherent) ([1](#_ENREF_1)), with the laser beating frequency tuned to the C-H stretching vibration mode at 2845 cm^-1^. The pump and Stokes beams are collinearly overlapped and combined, with the Stokes beam modulated at 2.3 MHz by an acousto-optic modulator (1205-C, Isomet). Images were taken on a lasing scanning microscope (FV300, Olympus) with a 60x water-immersion objective (NA = 1.2, UPlanApo/IR, Olympus). The acquisition time for a 512×512-pixel image was 1.1 second. The powers of laser beams were carefully adjusted and no photo-damages (e.g., blebbing of plasma membrane) to the tissues or cells were observed.

CARS imaging and spontaneous Raman spectral analysis from individual LDs were performed on a single platform as described previously ([2](#_ENREF_2)). A 5-picosecond laser at 707 nm was used as excitation beam for Raman spectral acquisition. Acquisition time for a typical spectrum from individual LDs was 20 s, with the beam power maintained around 15 mW at the sample. For each specimen, at least 10 spectra from individual LDs in different locations or cells were acquired.

**Quantitative analysis of SRS images and Raman spectra**

LD amount was quantified based on the SRS images using software ImageJ. Due to significantly higher signal level, LDs were picked up by “Threshold” function. The area fraction of LDs out of total area of tissues or cells were measured to quantize the LD amount. To analyze CE level, the background of Raman spectrum was removed as described ([2](#_ENREF_2)). CE level in individual LDs was quantified by analyzing the height ratio of the 702 cm^-1^ peak to 1442 cm^-1^ peak, which we showed to be linearly proportional to the percentage of CE out of total lipids (**Fig. S1b**). The percentage of CE out of total lipids was used to quantize the CE level.

**Cell viability, migration and invasion assay**

Cell viability was measured by Thiazolyl Blue Tetrazolium Blue (MTT) colorimetric assay (Sigma). Cell migration and invasion assays were performed in Transwell chambers (Corning) coated with and without Matrigel (BD Bioscience) respectively. 20% FBS and 0.2 ng/ml EGF were used as chemoattractant. 12 h (migration) or 24 h (invasion) after cells were seeded on top of the membrane, penetrating cells were stained with PI and counted under a confocal microscope.

**Cell cycle and apoptosis analysis**

PC-3 cells treated with 10 μM avasimibe for 3 days and the untreated ones were collected, fixed, and stained with 50 μg/ml propidium iodide (PI) at 37 °C for 30 min. The DNA content was measured by Cytomics FC500 flow cytometer (Beckman Coulter). Data were processed and analyzed by FlowJo software (Tree Star). Cell-cycle phases and frequencies of each phase were determined by fitting the data with the Watson-Pragmatic model. Annexin V/PI staining assay (Life Technologies) was used for detecting apoptotic cells by following manufacturer’s protocol.

**Fluorescence imaging of DiI labeled LDL uptake**

DiI labeled LDL was made in our laboratory following previously described method ([3](#_ENREF_3)). Cells were cultured in medium containing 10% lipodeficient serum with or without avasimibe treatment for 2 days. Then DiI-LDL was added to the medium at 50 µg/ml for 3 hours. Fluorescence images were acquired immediately after 3 h incubation using a confocal microscope. The LDL uptake was quantified using ImageJ by analyzing the area fraction of uptake LDL particles out of the total cellular area.

**Lipid extraction**

Lipid extraction from cells and tissues was performed according to Folch *et al* ([4](#_ENREF_4)). Tissue weights were measured and cell numbers were counted before lysis. Tissues (~ 50 mg) were cut and homogenized. Cell pellets or tissue homogenate was suspended in 200 µl PBS, and then 1.5 ml methanol and 3 ml chloroform were added. Vortex and incubate for 1 h on the shaker. 1.25 ml water was added and the mixture was vortexed, centrifuged at 1,000 g for 10 min. The lower phase was collected and dried under the flow of nitrogen. The extracted lipids were dissolved in acetone for further analysis.

**Electrospray ionization mass spectrometry (ESI-MS) and biochemical assay of CE and free cholesterol**

Lipids were extracted as described above. ESI-MS analysis was conducted according to the protocol described previously ([5](#_ENREF_5)). The relative intensity of CEs was normalized by cell numbers or by tissue weights. CE and free cholesterol were measured according to the manufacturer’s protocol (Amplex Red Cholesterol kit from Molecular Probes), and finally normalized by cell number or tissue weight.

**Immunoblotting**

After indicated treatments, cells were harvested and lysed in AMI lysis buffer (Active Motif) supplemented with protease and phosphatase inhibitor cocktail. Protein concentration was determined using the Bio-Rad protein assay kit. Protein extraction was subjected to immunoblotting with the antibodies against PTEN (Cell Signaling, 9188S), ACAT-1 (Santa Cruz, sc-69836), GRP78 (Santa Cruz, sc-13968), ATF4 (Cell Signaling, 11815), CHOP (Cell Signaling, 2895), SREBP-1 (Abcam, 3259), and β-actin (Sigma, A5441). β-actin was used as loading control for normalization.

**Immunohistochemistry**

Human pancreatic cancer and adjacent normal tissue were embedded in OCT (Tissue-Tek from Sakura Finetek USA) and cut into 6-μm frozen sections, followed by ice-cold 4% paraformaldehyde fixation for 10 min. Human tissue array sections were deparaffinized with xylene and rehydrated in an ethanol series. After blocking with horse serum, tissue sections were incubated with specific ACAT1 antibody (1:100, sc-69836 from Santa Cruz Biotechnology Inc.) overnight or ACAT2 antibody (1:200, A304-293A-T from Bethyl Laboratories Inc.) at 4 °C, followed by HRP-labeling anti-rabbit secondary antibody for 1 h at room temperature (MP-7401, Vector lab). The secondary antibody was revealed with 3.3’-diaminobenzidine (DAB) as substrate (SK-4105, Vector Lab) and counterstained with hematoxylin. The positive staining is brown color.

For TUNEL apoptosis assay, antigens were retrieved in antigen unmasking solution (Vector Laboratories) with a 2100-Retriever (PickCell Laboratories). Samples were used to TUNEL assay (Roche, 11684817910) according to manufacturer’s protocol. Fluorescent images were taken using Nikon A1R confocal microscopy under 40x objective.

**Transfection and gene knock-down**

Stable knock-down of ACAT-1 was conducted with ACAT-1 specific shRNA lentiviral transfection (Santa Cruz, sc-29625-V) according to manufacturer’s protocol. Scrambled shRNA lentiviral particles (Santa Cruz, sc-108080) was used as control. Transfected cells were selected under 2 µg/ml puromycin treatment for at least 10 days. Knock-down of SREBP1 and SREBP2 was performed using siRNA pLKO plasmids constructed previously ([6](#_ENREF_6)) with the targeting sequences CAACCAAGACAGUGACUUCCC and CAACAGACGGUAAUGAUCACG, respectively. Transfection of ACAT-1 shRNA plasmid (Sigma SHCLND-NM_003101), two PTEN shRNA plasmids (Santa Cruz, sc-29459-SH, and Sigma SHCLND-NM_000314), SREBP1/2 siRNA plasmids, and scramble shRNA plasmid (Santa Cruz, sc-108060) were performed using Lipofectamine® 2000 (Invitrogen, Cat# 11668-019) following the manufacturer’s protocol. Wild-type pLKO-PTEN and pLKO-GFP plasmid were gifts from Dr. Xiaoqi Liu and transfected using the Lipofectamine method.

**References**

1. Zhang D, Slipchenko MN, Cheng J-X. Highly Sensitive Vibrational Imaging by Femtosecond Pulse Stimulated Raman Loss. J Phys Chem Lett. 2011;2(11):1248-53.

2. Slipchenko MN, Le TT, Chen HT, Cheng J-X. High-speed vibrational imaging and spectral analysis of lipid bodies by compound Raman microscopy. J Phys Chem B. 2009;113(21):7681-6.

3. de Smidt PC, van Berkel TJC. Prolonged Serum Half-Life of Antineoplastic Drugs by Incorporation into the Low Density Lipoprotein. Cancer Res. 1990;50(23):7476-82.

4. Folch J, Lees M, Stanley GHS. A simple method for the isolation and purification of total lipids from animal tissues. J Biol Chem. 1957;226(1):497-509.

5. Liebisch G, Binder M, Schifferer R, Langmann T, Schulz B, Schmitz G. High throughput quantification of cholesterol and cholesteryl ester by electrospray ionization tandem mass spectrometry (ESI-MS/MS). Biochim Biophys Acta. 2006;1761(1):121-8.

6. Yue S, Li J, Lee S-Y, Lee Hyeon J, Shao T, Song B, et al. Cholesteryl Ester Accumulation Induced by PTEN Loss and PI3K/AKT Activation Underlies Human Prostate Cancer Aggressiveness. Cell Metab. 2014;19(3):393-406.

**Supplementary Figure Legends**

**Figure S1.** **CE accumulation in human pancreatic cancer tissues and cell lines (related to figure 1).** (A) Five Raman spectra taken from individual LDs in the same cell. Spectra from the same cell are very consistent with each other. (B) Calibration curve for quantification molar percentage of CE out of total neutral lipids. A linear correlation was established between the height ratio of the peak at 702 cm^-1^ and the peak at 1442 cm^-1^ and CE percentage, with the function Height ratio = 0.00255 × CE percentage (%). (C) Quantitative analysis of the amount of CE 18:2 in 5 pairs of normal and cancer tissues based on mass spectra. (D) Measurement of CE levels in 5 pairs of normal and cancer tissues using biochemical assays. (E) Representative SRS images of human pancreatic cell lines, including HPDE6, MIA PaCa-2, PANC-1, AsPC-1 and BxPC-3.

**Figure S2.** **CE accumulation in pancreatic cancer is mediated by ACAT-1 and expression of ACAT-1 correlates with poor patient survival** **(related to figure 2).** (A) Immunohistochemistry of ACAT-1 and ACAT-2 in the same pair of matched normal and cancer tissues. Representative images are shown. (B) Raman spectral measurement of CE levels in PANC-1 cells treated with DMSO (control) or 10 µM avasimibe for 2 days. (C) Representative mass spectra of CEs extracted from PANC-1 cells treated with DMSO (control) or 10 µM avasimibe for 2 days. (D) ACAT-1 was efficiently knocked-down by shRNA, confirmed by immunoblotting using antibody against ACAT-1. (E) Quantification of CE level in PANC-1 cells transfected with control shRNA or two ACAT-1 specific shRNAs. The quantification data are shown as means + SEM; n ≥ 10; ** *P* < 0.01.

**Figure S3. CE level in pancreatic cancer is regulated by PTEN and mediated by *de novo* cholesterol synthesis and LDL uptake (related to figure 3).** (A) PTEN was efficiently knocked-down by shRNA, confirmed by immunoblotting. (B) Overexpression of PTEN in MIA PaCa-2 cells was confirmed by immunoblotting. (C) Expression level of cleaved form SREBP1 was reduced by PTEN overexpression in MIA PaCa-2 cells. (D) Expression level of cleaved form SREBP1 was increased by PTEN knock-down in AsPC-1 cells. (E) Representative fluorescence images of DiI labeled LDL uptake in Mia PaCa-2 cells treated with DMSO (control) or 5 µM avasimibe. Red: DiI-LDL fluorescence imaging; gray: transmission imaging. Scale bar: 20 µm. (F) Quantification of area fraction of DiI-LDL out of the total cellular area. The data are shown as means + SEM; n = 8; *** *P* < 0.001.

**Figure S4.** **Inhibition of cholesterol esterification reduced pancreatic cancer cell proliferation, migration and invasion *in vitro* (related to figure 4).** (A) Viability assay of PANC-1 cells treated with avasimibe for 3 days. The data were fitted with a dose-response function using the software Origin8.5. (B) Cell proliferation assay of PANC-1 cells treated with DMSO or 10 µM avasimibe. The data are shown as means ± SD; n = 6; * *P* < 0.05, *** *P* < 0.001.

**Figure S5.** **Therapeutic effect of avasimibe in an orthotopic mouse model of pancreatic cancer (related to figure 5).** Hematoxylin and eosin (H&E) staining of tissues slices from tumor, liver, kidney, lung and spleen. A metastatic lesion (labeled “M”) in the liver of the control group was observed. Scale bar: 100 µm.

**Figure S6.** **ACAT-1 knock-down suppressed tumor growth and metastasis in an orthotopic mouse model of pancreatic cancer** **(related to figure 6).** (A) Body weight monitoring over time. The data are shown as means + SEM; n = 5 for control shRNA group, n = 6 for ACAT-1 shRNA group. (B) Representative SRS images of tumor slices. Scale bar: 10 µm.

**Figure S7.** **ACAT-1 inhibition increased cellular free cholesterol, induced ER stress and apoptosis (related to figure 7).** (A) Free cholesterol measurement in control or avasimibe treated tumor tissues. The data are shown as means + SEM; n = 3; ** *P* < 0.01. (B) Immunoblotting of GRP78 in MIA PaCa-2 cells transfected with control shRNA or ACAT-1 shRNA. (C) TUNEL assay of apoptotic cells in control or avasimibe treated tumor tissues. Apoptotic cells were stained as green, and nuclei were counterstained as blue. Representative images are shown. Scale bar: 50 µm.
