# Supplementary figures and images for: Abrogating cholesterol esterification suppresses growth and metastasis of pancreatic cancer
Source: Oncogene. 2016 May 2;35(50):6378–88. doi: 10.1038/onc.2016.168 (PMC5093084; doi:10.1038/onc.2016.168)

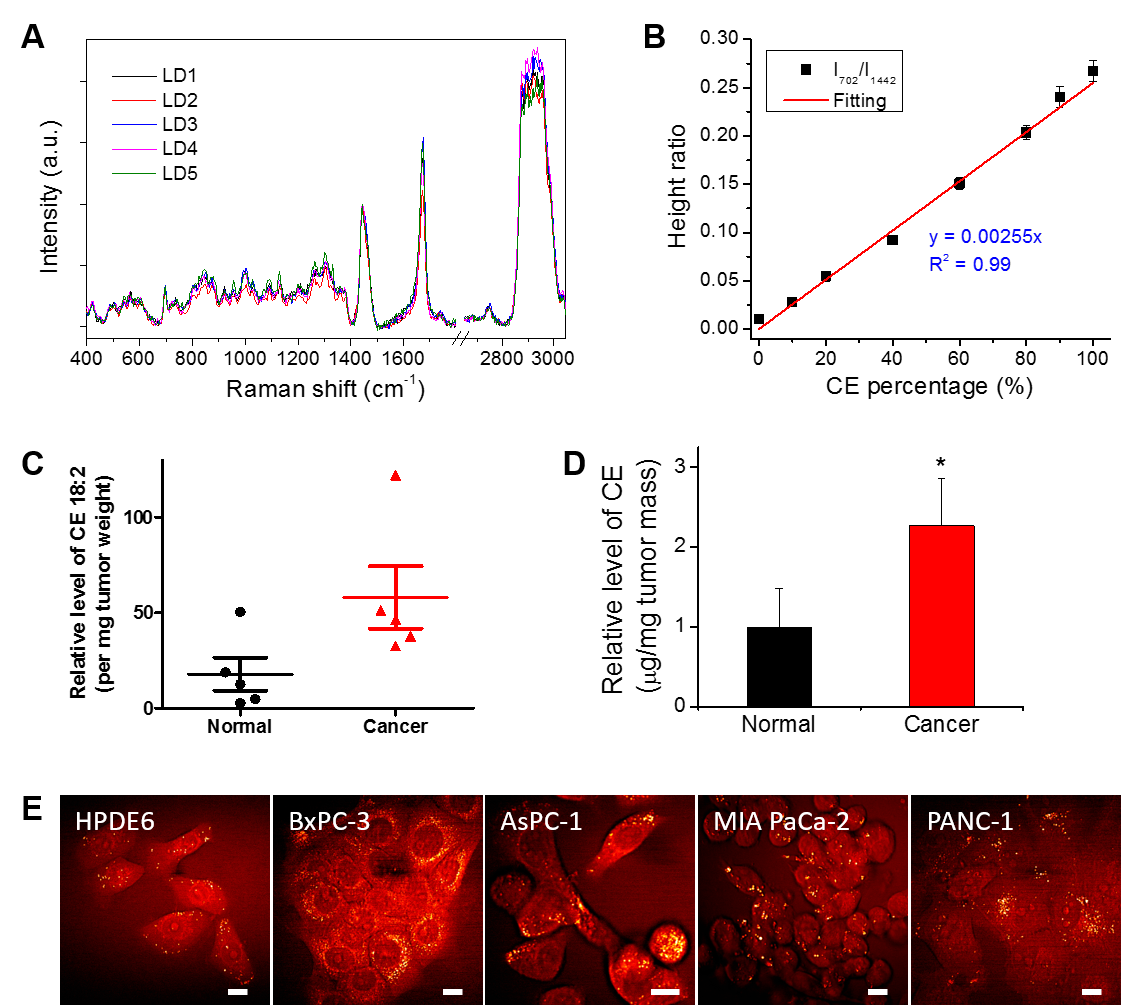

Supplement: Supplementary Figure [file onc2016168x2.png]

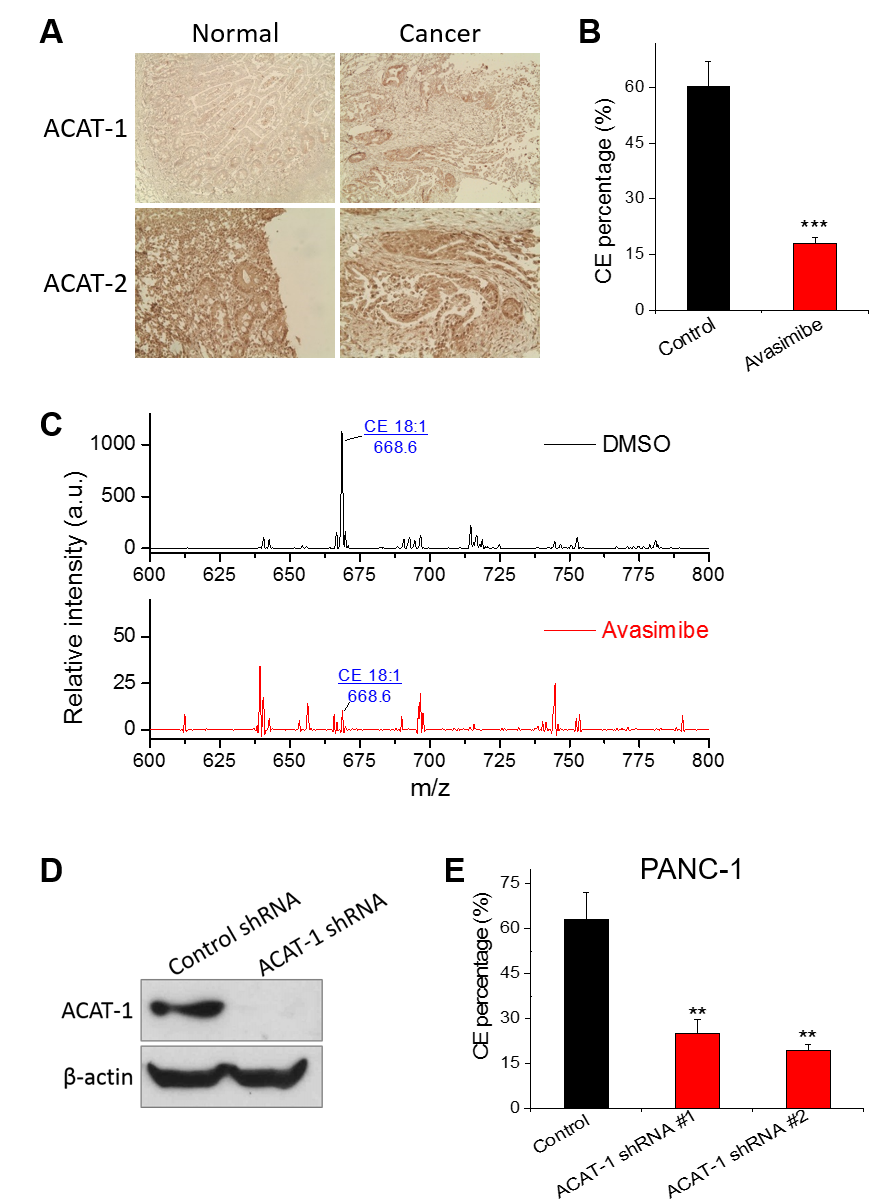

Supplement: Supplementary Figure [file onc2016168x3.png]

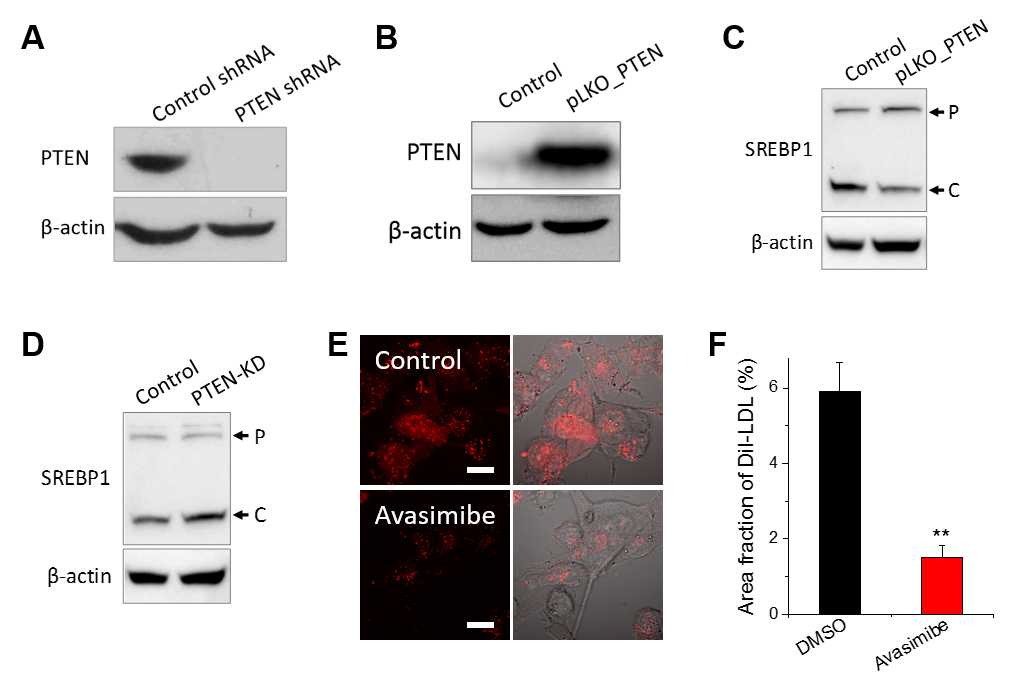

Supplement: Supplementary Figure [file onc2016168x4.png]

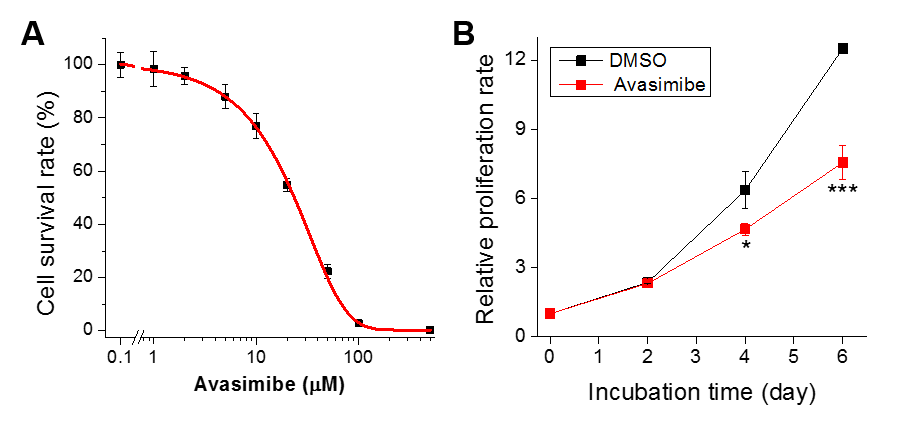

Supplement: Supplementary Figure [file onc2016168x5.png]

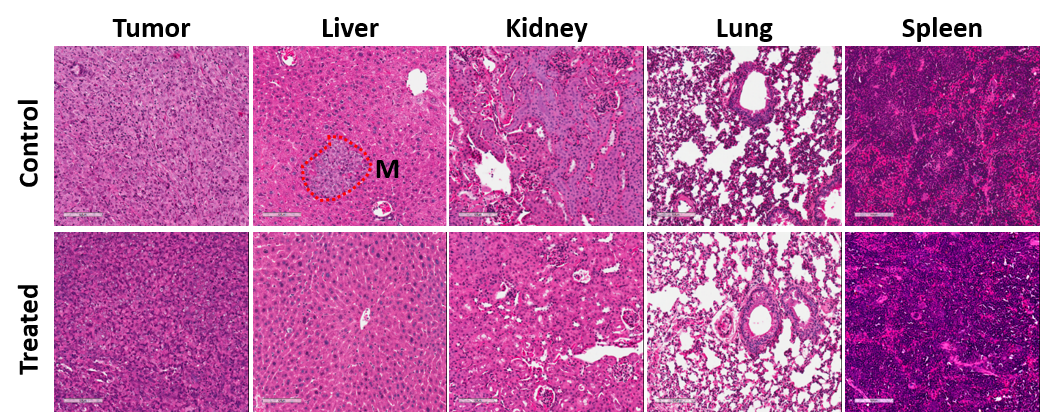

Supplement: Supplementary Figure [file onc2016168x6.png]

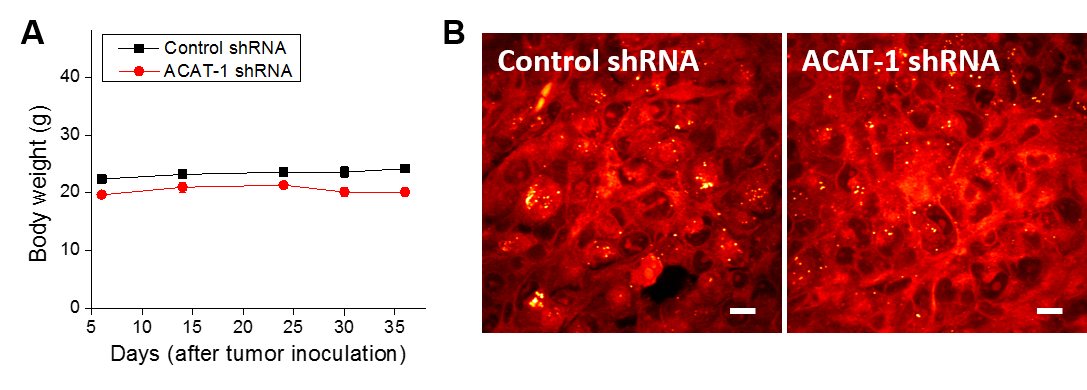

Supplement: Supplementary Figure [file onc2016168x7.png]

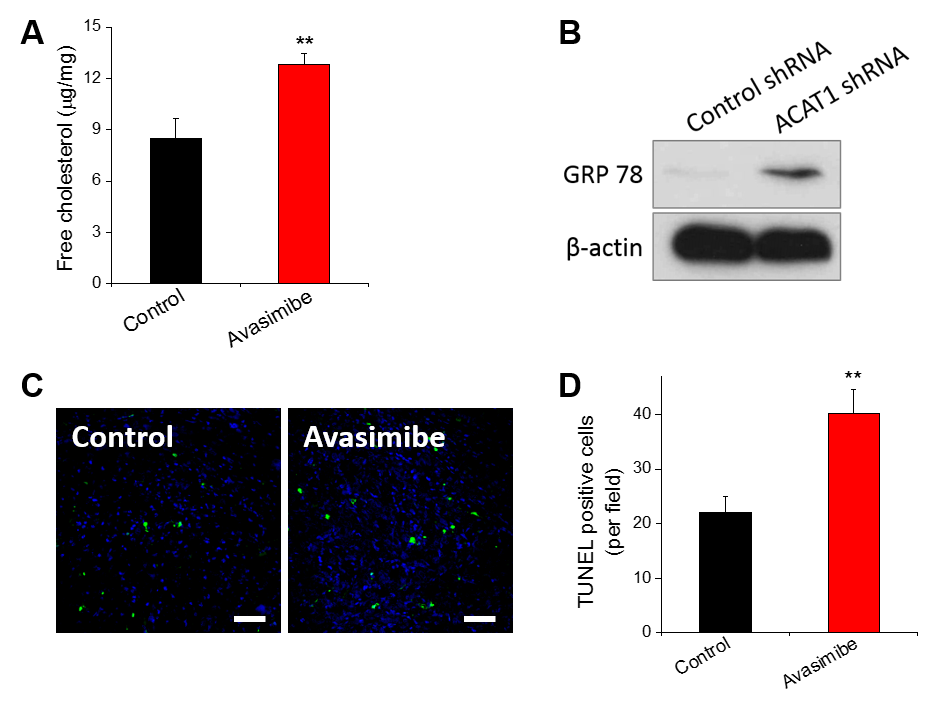

Supplement: Supplementary Figure [file onc2016168x8.png]
